# Delineation in fuzzy boundaries: Canonical ordination analysis discriminates between cryptic species by linking taxonomy to genetics, morphology, climate, and space

**DOI:** 10.1371/journal.pone.0334617

**Published:** 2025-10-31

**Authors:** Ioan Sîrbu, Ana Maria Benedek, Sebastian Hofman, Aleksandra Jaszczyńska, Andrzej Falniowski

**Affiliations:** 1 Biology and Ecology Research Center, Faculty of Sciences, Lucian Blaga University of Sibiu, Sibiu, Romania; 2 Doctoral School of Engineering Sciences and Mathematics, Lucian Blaga University of Sibiu, Sibiu, Romania; 3 Department of Comparative Anatomy, Institute of Zoology and Biomedical Research, Jagiellonian University, Kraków, Poland; 4 Department of Malacology, Institute of Zoology and Biomedical Research, Jagiellonian University, Kraków, Poland; 5 Department of Invertebrate Evolution, Institute of Zoology and Biomedical Research, Jagiellonian University, Kraków, Poland; Laboratoire de Biologie du Développement de Villefranche-sur-Mer, FRANCE

## Abstract

In species complexes, delineation of cryptic species remains a major challenge due to their morphological similarities despite significant genetic divergence. The related problems of defining and recognizing hidden diversity impact a range of sciences, posing theoretical and practical problems. Our article introduces a novel application of canonical ordination analysis as a powerful tool for examining and testing the interplay between genetic and morphological variability in cryptic species while accounting for their relation to climate and spatial descriptors, aiming to discriminate them by integrating multiple sources of predictors. We used data on 60 *Fruticicola* sp. populations across its range, belonging to three cryptic species—*Fruticicola fruticum*, *F. similis*, and *F. gemina*. We used five variable categories: taxonomy (response variables), genetics and morphology (response variables and predictors), climate and space (predictors). We applied distance-based redundancy analysis and canonical correspondence analysis to examine and test relationships among these variable categories, variation partitioning procedure to disentangle the effects of the considered predictors, and linear discriminant analysis to test their discriminatory power. Morphology was best explained by climate (mainly humidity), whereas genetic distances showed patterns shared between climate and space. Because the three pseudocryptic species were defined on molecular basis, taxonomy was almost completely explained by genetic distances. Although, when considered alone, morphology, climate, and space did not perform well in discriminating the species, when included together in the models, they were able to correctly classify the samples. We demonstrate how this multivariate canonical approach enhances species delimitation, offering a clearer understanding of cryptic diversity and its ecological implications. By linking these facets, we provide a comprehensive framework that connects taxonomic classifications with ecological and evolutionary processes. Hence, our results bring more insight into the processes linked to hidden diversity while providing new tools for its assessment, broadening the framework for applied research.

## 1. Introduction

The challenge of defining, recognizing, and delineating cryptic species and addressing other species complex issues presents both theoretical and practical difficulties. These challenges impact evolutionary biological studies, biodiversity assessment and conservation programs, ecological research, environmental management, and many others [1,2]. Understanding evolutionary processes at a specific level requires balancing and intimate understanding of two opposing phenomena: genetic variation without (or with nonobvious) phenotypic distinct expression, which may lead to and define cryptic species, versus genetic uniformity or isogenic populations showing heterogenous phenotypes, defining the phenotypic plasticity or noise [3]. With rising accessibility to molecular methods applied in phylogenetics paralleled by dropping methodological costs, the number of studies concerning this subject and newly described species, split from one or a few formerly recognized taxa, has increased dramatically in recent years. This process may cause havoc even in conservation legislation on a continental or even world scale. A recent example is the case of the freshwater bivalve *Unio crassus* Philipsson, 1788, a species of community interest listed in Annexes II and IV of the EUHSD (92/43/EEC 1992)—the Habitats Directive, which proved to be a complex of 12 distinct species [4]. Some of these species are widespread, others have a limited range, and all are subject to different types and degrees of pressure and dangers, possibly classified in different vulnerability categories on the red list. Adapting EU and member countries’ legislation to accommodate these species is one part of the problem, while recognizing them in the field is another one because, to the best of the present-day knowledge, their differentiation can be done with certainty and only for sympatric taxa, by molecular methods. Issues related to the species complex, to whom cryptic species belong, are many: identification, especially during field studies, biodiversity assessment, protection, monitoring, management, and ecology, among others, and they fuel highly controversial outcomes. For instance, the non-random frequency across taxonomic groups and types of habitats was challenged, some arguing that, when corrected for species diversity and number of studies, cryptic species may be more evenly distributed among major metazoan phylogenetic lineages and biomes [5], while others contradict this assumption stating that cryptic diversity among metazoan phyla varies by up to two levels of magnitude [6]. An estimate suggested that for each insect species described on a morphological basis, there are, on average, 3.1 cryptic species. Thus, the unknown projected species richness soars accordingly [7]. Cryptic species have been reported among many biological groups and are frequently supposed to be a large part of extant biodiversity [5,8,9]. DNA “barcoding”, as reviewed by DeSalle and Goldstein [10], and Janzen *et al.* [11], followed by meta-barcoding, environmental genomics, and others, resulted in the discovery of numerous species whose morphology was not distinct.

There are many publications concerning cryptic species in land pulmonates [12–19]. In our molecular study on 60 local populations of *Fruticicola* Held, 1838 (Family Camaenidae Pilsbry, 1859), widely distributed within the range of *Fruticicola fruticum* (O. F. Müller, 1774) [20], we discovered two species that we classified as pseudocryptic, following the terminology of Mann and Evans [21]. They are distinct molecularly, but their genital morphology differs only slightly. However, their shells’ biometry was not studied. There is substantial literature on shell color and banding polymorphism in *F. fruticum* [22–25]. The inheritance of banding patterns seems to vary among species and is more complex than in other land species, such as *Cepaea* [26–28]. On the other hand, interpopulation differences in shell biometry are poorly studied [20,26]. Gastropod shell proportions have been studied for a long time [29–31 see 32]. However, in the Helicoidea, like in other terrestrial pulmonates, there are numerous publications reporting the shell size [33–39] and shell wall thickness [37,40–43], but much less about shell proportions [34,36,41,44–52].

The genetic p-distances, and genetic distances in general, reflect genetic isolation among local populations. In isolation by distance and stepping stone models of interpopulation divergence, these distances are associated with the geographic distance [36,53–55]. Additionally, they reflect differences in population genetic structure, which are influenced by local environmental conditions.

**Fig 1:**
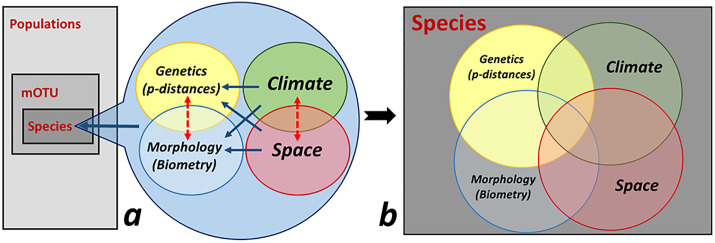
The aim of this work is a) to relate genetics and morphology to climate and spatial descriptors and each of these to variability in the populations, mOTU, and cryptic species, followed by b) a variation partitioning between the four classes of predictors used in the cryptic species discrimination. **a)** Blue arrows suggest asymmetrical relations (regression *s. lato* or canonical ordination analysis) between levels of predictors, while dotted red arrows indicate dependent relations among intertwined classes of explanatory variables. **b)** The Venn diagram highlights the unique and shared effects (fractions of explained variation) of the four categories of predictors used in the discrimination of the cryptic species (response variables).

Our study aims to relate variability in, and discrimination of, populations, mOTUs (molecular Operational Taxonomic Units), and species as responses to four classes of predictors, namely genetics (p-distances between populations), morphology (biometric descriptors), climatic parameters, and spatial descriptors. We then examine relationships among these four classes of explanatory variables using regression (*sensu lato*) and canonical ordination methods, concluding with a variation partitioning between all classes of explanatory variables used to discriminate the cryptic species (Fig 1). Although various studies examine the relationships among categories of variables aforementioned, none addresses the interdependence of all these facets of the ecological and evolutionary processes driving speciation and here, we aim to fill this gap. By this, our work brings more insight into cryptic species discrimination and the interplay of variability (genetic and morphological) with external drivers (climate and space). We show that a selection of morphology, climate, and space predictors might well discriminate between cryptic species, in the same degree as genetic features, when using appropriate multivariate canonical methods.

## 2. Materials and methods

### 2.1. Samples and variables

This study is based on 249 gastropods of *Fruticicola* sp. sampled from 60 sites from its entire range, belonging to 8 mOTU (codified with the letters from A to H), and three pseudocryptic species [20], denoted as I – *F. fruticum*, II – *F. similis*, and III *– F. gemina*. The sampling sites covered an area representative of the species’ range, including western, central, eastern, and southeastern Europe. Snails were hand-collected by several contributors, including the authors, from different European countries (Fig S1 in S1 Supporting Information) by a simple random sampling design from specific habitats. Each sampling site is identified by latitude and longitude (decimal degrees, WGS 84).

The animals were fixed in 80% ethanol, changed during the first hours after collection, and afterwards stored at −20 degrees Celsius. The shells of 183 specimens have been photographed (with a Canon EOS 50D camera with MACRO MPE 65 lenses and MR14EX Macro Ring Light), dissections being done under a NIKON SMZ-18 stereoscope microscope.

In the database the location of each sampling site was codified numerically (from 1 to 60), with its latitude (denoted Y), longitude (X, expressed in decimal degrees), and altitude (denoted Alt, in meters a.s.l.). Genetic analysis was based on the previously published p-distances obtained from phylogenetic analysis of COI sequences (cytochrome oxidase c subunit I) and from mitochondrial ribosomal 16S [20]. We sequenced 125 specimens from all 60 sampling sites across Europe (Fig S1 in S1 Supporting Information). Uncorrected p-distances, the proportion of nucleotide sites at which two sequences being compared are different [56], were calculated in MEGA 7 [57].

**Fig 2:**
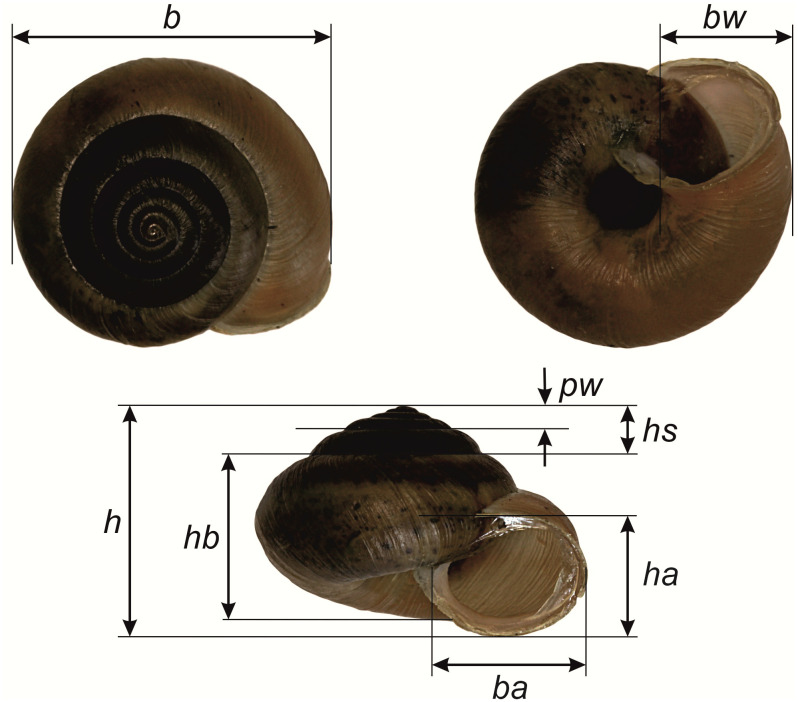
Morphological (biometric) shell parameters used in this study. Shell measurements: b—shell breadth, ba—aperture breadth, bw—body whorl width, h—shell height, hb—body whorl height, ha—aperture height, hs—spire height, pw—penultimate whorl width.

For the morphometric analysis, we measured eight shell parameters ([20], Fig 2) in 183 adult snails (with completely developed lips, which were also photographed) from 24 sampling sites (Fig S1 in S1 Supporting Information). The number of measured individuals per sampling site ranged between one and 38, with a mean of 7.63 and a standard error of 1.77. One more parameter—the ratio of width to height (b/h)—was added. Shell measurements were taken with an electronic caliper and expressed in mm, except for the b/h ratio, which is nondimensional.

During the study all applicable institutional and national guidelines for the care and use of animals were followed. All aspects of fieldwork and animal handling complied with EU Council Directive 86/609/EEC on the experimental use of animals. The working protocol was also approved by the Ethical Commission for Scientific Research (approval 1/20.06.2022) of the Lucian Blaga University of Sibiu.

As climatic variables, we used 21 parameters for each of the 60 locations: average precipitation in January (Rjan), April (Rapr), July (Rjul), October (Roct), total amount of annual precipitation (Rtot)—all in mm, precipitation coefficient of variation (Rcv), number of wet days in January (Wjan), April (Wapr), July (Wjul), October (Woct), and in the whole year (Wet), relative humidity in percentage (RHum), average duration of annual sunshine in percentage of possible (Sunt), average temperature for January (Tjan), April (Tapr), July (Tjul), October (Toct), and for the whole year (Tavg), average maximum temperature in the hottest month (Tmax), minimum temperature in the coldest month (Tmin), and the number of months with minimum temperatures above 10°C (Tno10); all temperature values are in degrees Celsius. Climatic parameters were obtained from AQUASTAT Climate Information Tool [58].

### 2.2. Statistical methods and software

Canonical ordination methods have been used to link, explain, and test the relations between response variables (RV) and predictors (explanatory variables, EV). As response matrices, we considered taxonomy (species and mOTU). Morphology (biometric descriptors) and genetics (matrix of p-distances) were included as RV in some analyses (in relation to climate and space) and EV in others (explaining taxonomy). The species and mOTU factorial variables were transformed into dummy variables, each level (species or mOTU) becoming a binary descriptor (with values of 1 and 0). Thus, the response matrix consisted in three and respectively eight variables. The biometric variables of the populations and separately—considering the mean values—of the mOTUs, were also handled as RV.

In analyses, we used the available data on all 60 sampling sites, except those involving morphology (including the final variation partitioning), because biometric descriptors were available only for 24 sites (Fig S1 in S1 Supporting Information).

For short gradients, we used linear methods, namely redundancy analysis (RDA) for rectangular matrices and distance-based RDA (db-RDA) for distance matrices (i.e., genetic distances between populations or mOTU). For dummy variable RVs, we used the canonical correspondence analysis (CCA). We estimated and tested the simple terms effects (as if the variable would act as a singular predictor) and conditional terms effects (explanatory power of predictor after accounting for the variability explained by those already selected). We considered the best model explaining the variation in the RV, the most parsimonious one, obtained by interactive forward selection of predictors, including only significant predictors in terms of their conditional effect. The selection was done in descending order of explained variance and by the adjusted p-values. Significance was tested by the Monte Carlo permutation test with a 999 unrestricted permutations scheme. For multiple testing (i.e., simple and conditional effects of individual variables), probabilities adjusted (p-adj) by the false discovery rate (FDR) are reported. In direct ordination methods, we report the percentage of variation in RV explained by the EV as adjusted explained variation (R^2^-adj). In all RDA, the response variables were centered and not transformed.

To test the significance of specific relations between one particular RV and the available EV, generalized additive models (GAM) were used, using 3 degrees of freedom (df) as term smoothness, a Gaussian response distribution, and a stepwise selection using the Akaike criterion (AIC). Only additive models with an AIC value lower than the null models have been kept and reported.

To disentangle the variation in RV (i.e., taxonomy, genetics, morphology) explained by two or more groups of EV, we performed the variation partitioning procedure. Spatial trends were detected using the geographical gradients separately as predictors and also coordinates polynomials (X, Y, X^2^, Y^2^, X^3^, Y^3^, XY, XY^2^, and X^2^Y), also called trend surface polynomials [59] used as EV, with a forward selection procedure in RDA. These methods are facile to interpret and show the relative importance of the geographic coordinates, but they model only monotonic or simple (unimodal to bimodal) relations between RV and EV. Therefore, we decided to use additionally the PCNM (Principal Coordinates of Neighbor Matrices) variation partitioning procedure to quantify the spatially structured and non-structured fractions of the variability explained by climatic parameters and of variation in genetic distances and morphology that are not related to climate, but to space patterns. The PCNM [60] is a particular method of the more general db-MEM (distance-based Moran’s Eigenvectors Maps *in* [61]). It is embedded in Canoco 5.15 [62], allowing us to import a distance matrix, what we did for the genetic distances and their relations to the spatial eigenvectors, and for the variation partitioning. However, in Canoco variation partitioning templates, the response variables data tables are rectangular (serving as basis for either an RDA or a CCA), which is not the case with the genetic distances. Therefore, we first summarized the variability in the p-distance matrix by a Principal Coordinates Analysis (PCoA). Then, the scores on the PCoA axes served as response variables, used in a variation partitioning analysis, first with simple, then with conditional terms effect.

The analyses concluded with a variation partitioning between the four classes of predictors (selected biometric and climatic descriptors, PCoA axes obtained on the genetic p-distances, and the corresponding PCoA axes scores from the db-MEM analysis on geographical coordinates) involved in discriminating among the three pseudocryptic species. We have also tested the ability of the selected descriptors, other than the genetic p-distances, to delineate between the cryptic species by means of linear discriminant function analysis (LDA). We included as predictors the variables that had a significant explanatory power in the CCA relating the mOTU classification separately to morphology, climate, and space. We used Wilks’ lambda, its conversion to F-value, and corresponding p-value for testing the discriminatory power of the model. In addition, we also reported for each variable in the model its significance when included as a single predictor (simple effect) and when comparing the models with and without that variable (partial effect). We tested the significance of the two LDA axes by one-way ANOVA (assumptions of normality and homoscedasticity were met) using the samples’ scores on each axis as responses and the three species as explanatory variables. Because of the small sample size and large number of predictors, we accounted for the potential overfitting of the model. The sample size was too low to allow cross-validation by dividing our data into a training and test dataset; therefore, we adopted the jackknife resampling approach. We constructed 24 models, leaving one sample out each time to be subsequently classified using the others in the LDA, and then checked the percentage of correct assignments. Ordination analysis and other uni- (GAM) and multivariate methods were done in Canoco 5.15 [62]. The variation partitioning between two classes of predictors was computed in Canoco and illustrated using Venn diagrams, whereas for four categories of predictors, we applied functions in the R package “rdacca.hp” [63] to calculate variation parts and “nVennR” [64] and “UpSetR” [65] to illustrate them. LDA and associated testing were performed using packages “MASS” [66], “Morpho” [67], and “klaR” [68]. We used R versions 3.6.1 and 4.3.0 [69].

## 3. Results

### 3.1. Analysis of morphological variability in populations explained by spatial and climatic predictors

The response variables matrix consisted of 24 populations for which biometric variables were available. As predictors, we first considered each geographical gradient separately. There was no significant relation between the altitude and the biometric variables (RDA with all and GAM with each morphological descriptor proved insignificant). The latitudinal (Y) gradient explained 17.25% of the (adjusted) variation in the shell morphology (i.e., all biometric variables) variation (pseudo-F = 5.8, p = 0.019). In contrast, there were no significant overall relationships between longitude (X) and the shell morphology.

Exploring the relationships between shell morphology and trend-surface polynomials of spatial variables, several significant models have emerged. One of the best models explaining the overall variation was XY^2^ (p = 0.035) and Y^3^ (p = 0.045), the adjusted explained variation being 22.29%. This means that selected polynomial spatial gradients explained about one-quarter of shell morphological variability.

**Fig 3:**
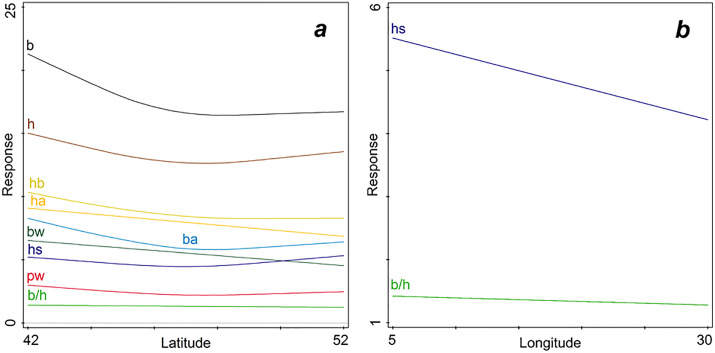
GAM between the biometric (morphological) shell descriptors (Response) and a. Latitude and b. Longitude. Codes of biometric variables are given in the text (Material and Methods section).

Testing for pairwise relations between either latitude or longitude and responses in individual morphological descriptors resulted in significant GAMs. All nine biometric descriptors were significantly related to latitude (only hs had a marginally significant relation). Some decreased monotonically with increasing latitude (bw, ha, b/h), the others by a quadratic model (Fig 3a, Table S1 in S1 Supporting Information). In the GAMs with longitude as a predictor only hs and b/h were marginally significantly (0.05 < p < 0.1) and negatively linked (decreasing with increased longitude at the European scale), thus the spire was higher, and the general shape of the shell was flatter towards the West (Fig 3b, Table S2 in S1 Supporting Information).

**Fig 4:**
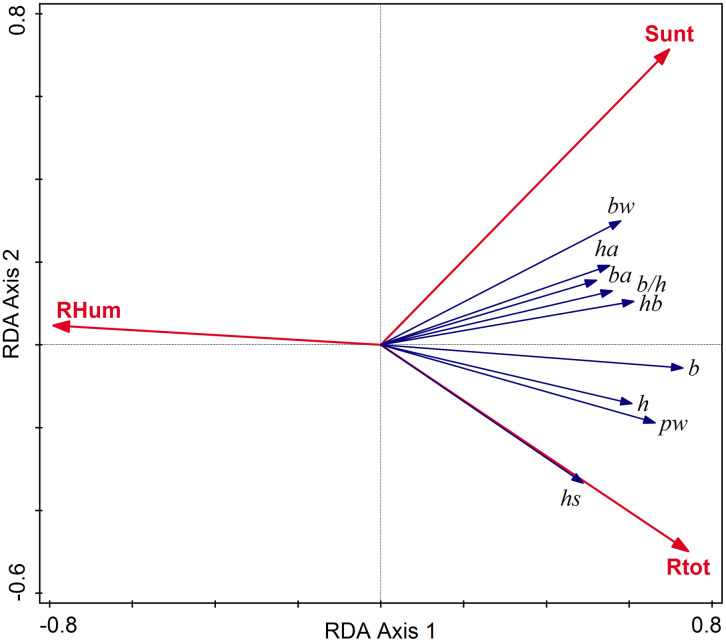
RDA ordination diagram with biometric variables predicted by RHum (relative humidity), Rtot (total amount of annual precipitations in mm), and Sunt (average duration of annual sunshine in percent of possible). The model is significant (p = 0.003, R^2^-adj = 35.7%). Codes of biometric variables (blue arrows) are explained in the text.

In the RDA with biometric variables in relation to climatic descriptors, the humidity (rain, wet days) had the major effect, considering the simple term effects. Rainfall in January (Rjan) and October (Roct) had a significant predictive power, each explaining about 31% in the variation of morphology (p = 0.042). Marginally significant (0.05 < p < 0.06) were in descending order of the explained variation the effects of the: relative humidity (RHum, r^2 ^= 26.1%), total rain (Rtot, r^2 ^= 23.4%), rain in April (Rapr, r^2 ^= 23%), average duration of the annual sunshine in percent of the total possible (Sunt, r^2 ^= 21.2%), and wet days in July (Wjul, r^2^ = 18%). We entered this selection of predictors in the interactive forward selection procedure for the RDA of the joint effects of climatic variables. The best model, including RHum, Rtot, and Sunt (pseudo-F on all axes = 5.3, p = 0.003), explained 35.7% of the shell morphology variation. All the biometric variables were directly related to the Sunt and Rtot, and negatively linked to RHum (Fig 4). A competing best model included only Rjan (R^2^-adj = 28.28%).

#### 3.1.1. Variation partitioning between climatic and spatial predictors’ effects on morphological (biometric) variability.

**Fig 5:**
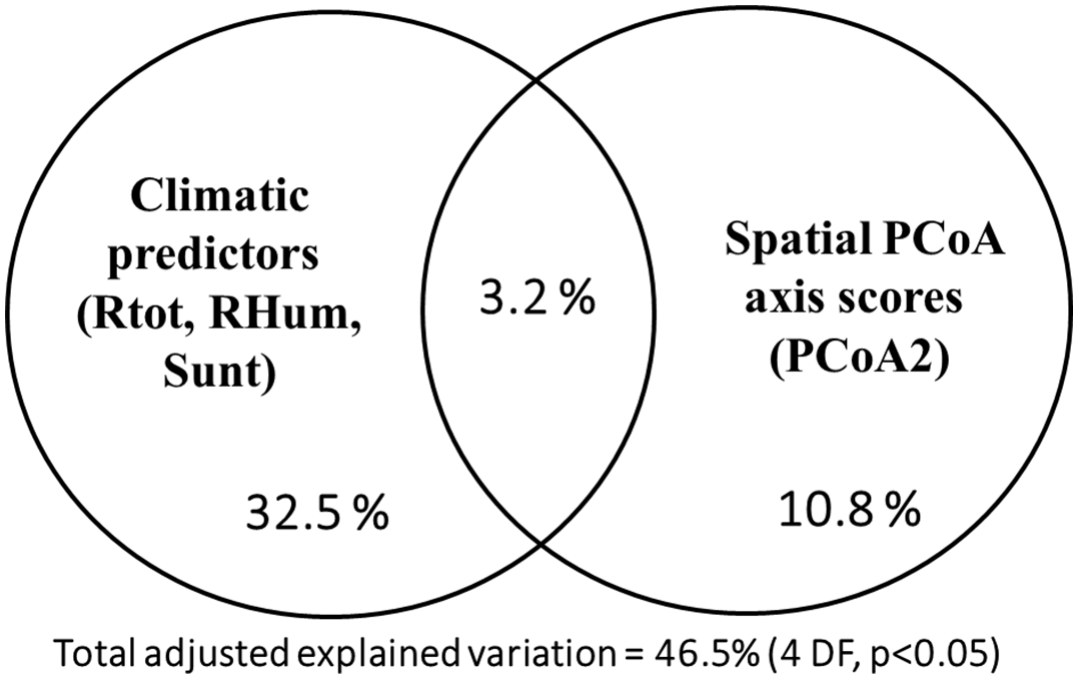
Venn diagram showing the variation partitioning of the morphological (biometric) variability in the *Fruticicola* populations explained by the selected spatial eigenfunction and climatic predictors.

We have done a variation partitioning between the effects of spatial eigenvectors (by a PCNM) and climatic parameters, investigating their predictive features on biometric response variables, the explanatory variables being selected by an interactive forward procedure. Among the climatic parameters Rtot, RHum, and Sunt, and among the spatial eigenfunctions, only PCoA2 axis scores were significant, explaining 46.5% of the morphological variability. Climatic features (unique effect 32.5%) had a greater effect on morphological variability than the spatial predictors (unique effect 10.8%), while their shared effect was comparatively small (3.2%) (Fig 5). Both simple and conditional effects of the predictor groups were significant (p < 0.05).

### 3.2. Genetic p-distances explained by spatial and climatic predictors

In the db-RDA between the p-distances matrix of the 60 populations and the trend-surface polynomials of Latitude (Y) and Longitude (X), the adjusted explained variation was 69.4%; the selected and significant terms were Y^2^ (quadratic latitude, explaining 64.9% of the variability in p distances), followed at a lower order of magnitude by X^3^ (cubic longitude, explaining 3.3%). Thus, genetic distances are highly and significantly explained by the spatial gradients, the latitude being much more important (about 90% in relative terms) compared to longitude; the polynomial terms of coordinates explained about two thirds of the maximum variation in p-distances between the populations. We used altitude as the single explanatory variable in another db-RDA and have learned that it explained 23.2% of the p-distances variation (pseudo-F = 18.8, p = 0.001). Compared to latitude (if considered as such, latitude explained 65.4%), altitude plays a minor role, but not insignificant if considered alone, in explaining the genetic distances between the populations.

In the variation partitioning of genetic distances between altitude and spatial eigenvectors, the overall adjusted explained variability in PCoA axes scores of the p-distance matrix was 82.1%. Of this, the unique effect of the selected spatial eigenfunctions was 58.9%, and the rest (23.2%) was shared between the predictor groups, with altitude having no unique effect. Thus, when considered together with the spatial coordinates, altitude was of no importance in explaining genetic distances between populations.

The overall null hypothesis that the climatic parameters are not related to the p-distances between the 60 studied populations was rejected by means of a db-RDA analysis (test on all axes pseudo-F = 12.0, p = 0.001), the adjusted explained variation being 79.6%. Thus, the climatic parameters may explain almost 80% of the variation in p-distances.

Testing for the simple and conditional effects of the climatic predictors led to the selection of parameters characterizing wet days, humidity, and rain but not related to temperature (Table 1).

**Table 1:** Simple and conditional term effects of climatic predictors on the p-distances between populations, within a db-RDA analysis. p-adj – probabilities adjusted by the false discovery rate. Codes of all climatic variables are explained in the Material and methods section.

Simple term effects					Conditional term effects				
Predictor	Explains %	pseudo-F	p	p-adj	Predictor	Explains %	pseudo-F	p	p-adj
Rapr	31.3	26.5	0.001	0.0021	Rapr	31.33	26.5	0.001	0.00525
Woct	31.2	26.4	0.001	0.0021	Rtot	28.76	41.1	0.001	0.00525
Sunt	30.6	25.6	0.001	0.0021	Wjul	6.74	11.4	0.001	0.00525
Wjul	28.2	22.8	0.001	0.0021	RHum	5.55	11.1	0.001	0.00525
Rcv	27.6	22.1	0.001	0.0021	Wjan	1.95	4.1	0.005	0.01313
Wjan	26.5	21	0.001	0.0021	Wapr	2.8	6.5	0.002	0.0084
Wet	23	17.3	0.001	0.0021	Wet	1.9	4.7	0.004	0.01313
Rjan	21.1	15.6	0.001	0.0021	Roct	1.74	4.6	0.005	0.01313
Roct	16.2	11.2	0.001	0.0021	Rcv	1.09	3	0.021	0.0483
Rtot	14.2	9.6	0.001	0.0021	Tapr	0.97	2.8	0.051	0.09736
RHum	8.9	5.7	0.012	0.02291	Tno10	1	3	0.023	0.0483
Wapr	8.1	5.1	0.014	0.0245	Tmax	0.54	1.6	0.167	0.29225
Tjan	3.6	2.2	0.133	0.21485	Tjul	0.4	1.2	0.273	0.4215
Tmin	2.3	1.4	0.215	0.301	Tavg	0.39	1.2	0.281	0.4215
Tjul	2.2	1.3	0.211	0.301	Sunt	0.37	1.1	0.326	0.4564
Rjul	2.2	1.3	0.255	0.33469	Rjan	0.34	1	0.372	0.48825
Tno10	0.9	0.5	0.547	0.67571	Toct	0.27	0.8	0.511	0.63124
Toct	0.8	0.4	0.627	0.69853	Rjul	0.23	0.7	0.611	0.71283
Tavg	0.7	0.4	0.632	0.69853	Woct	0.18	0.5	0.715	0.75075
Tapr	0.4	0.3	0.8	0.8	Tjan	0.1	0.3	0.882	0.882
Tmax	0.4	0.2	0.796	0.8	Tmin	0.21	0.6	0.662	0.73168

The single predictor having a significant simple term effect, not directly related to rainfall and wet days was Sunt (average duration of annual sunshine in percentage of all possible). Still, its effect was removed by better predictors in the final model, where the conditional term effects were considered. In the final model the best predictors were, in descending order, the average precipitations in spring (Rapr, explaining 31.3%), the total amount of annual precipitations (Rtot, 28.8%), all the remaining predictors showing much lower explanatory power—wet days in July (Wjul, 6.7%), relative humidity (RHum, 5.6%), followed by the number of wet days in winter (Wjan), spring (Wapr) and along the whole year (Wet), the average precipitations in October (Roct), and the precipitations’ coefficient of variation (Rcv). The db-RDA diagram is given in Fig S2 in S1 Supporting Information, with the selected significant predictors (p < 0.05) and populations’ axes scores plotted in ordination space, the adjusted explained variation being 78.6% (p = 0.001 for both first and all axes). Two climatic predictors related to temperature, not included in the best model, had significant conditional term effects —number of months with temperatures above 10°C (Tno10)—and marginally significant—average April temperature (Tapr) (Table 1), each explaining about 1% in p-distance variation.

#### 3.2.1. Variation partitioning of p-distances between climatic and spatial gradients.

**Fig 6:**
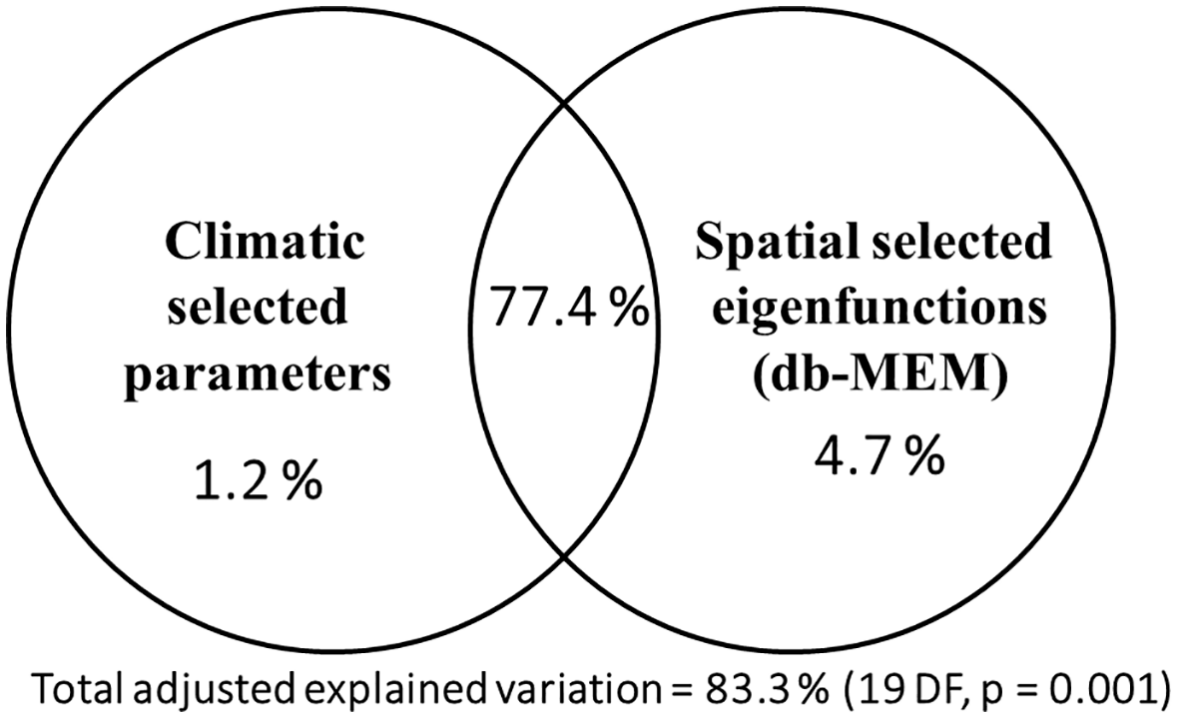
Variation partitioning db-MEM with the forward selection of climatic parameters and spatial eigenvectors explaining the variability in p-distances among the 60 *Fruticicola* populations.

The total variation in p-distances (summarized by their PCoA scores on all axes with positive eigenvalues) explained by selected variables was 83.3%, most part being their shared effect (77.4%), whereas the unique effect of the climate was 1.2% and of the spatial eigenfunctions 4.7% (Fig 6). The climatic parameters selected by the forward selection procedure, were: Rapr, Roct, Rtot, Rcv, Wjan, Wapr, Wjul, Wet, and RHum, and the PCoA scores axes for the spatial eigenvectors were PCoA1, 2, 4–9, 11, and 16, indicating both large-scale (small values axes) and small-scale spatial effects (larger axes, e.g., PCoA16) on genetic distances variability. The tests of single effects fractions were significant (p = 0.001).

Thus, a very large part of the variability in p-distances among populations was explained by both spatial and climatic parameters (more than 80%), their shared effect being largely overlapping, while the unique effects were relatively small.

### 3.3. Classification of populations into mOTUs and species explained by biometric variables

To search for discriminant patterns between the classes, we used a CCA with the mOTUs as response and the biometric variables as predictors. The adjusted explained variability in the mOTU classes by all biometric variables was 22.1%, the test on all axes being significant (pseudo-F = 1.7, p = 0.004). Four predictors had significant (p < 0.05) simple term effects, namely pw, b, bw, and hb, whereas the rest were marginally significant (0.05 < p < 0.1). The conditional term effects were significant only for pw, and marginally significant for b and hb; all the others were discarded from the model.

**Fig 7:**
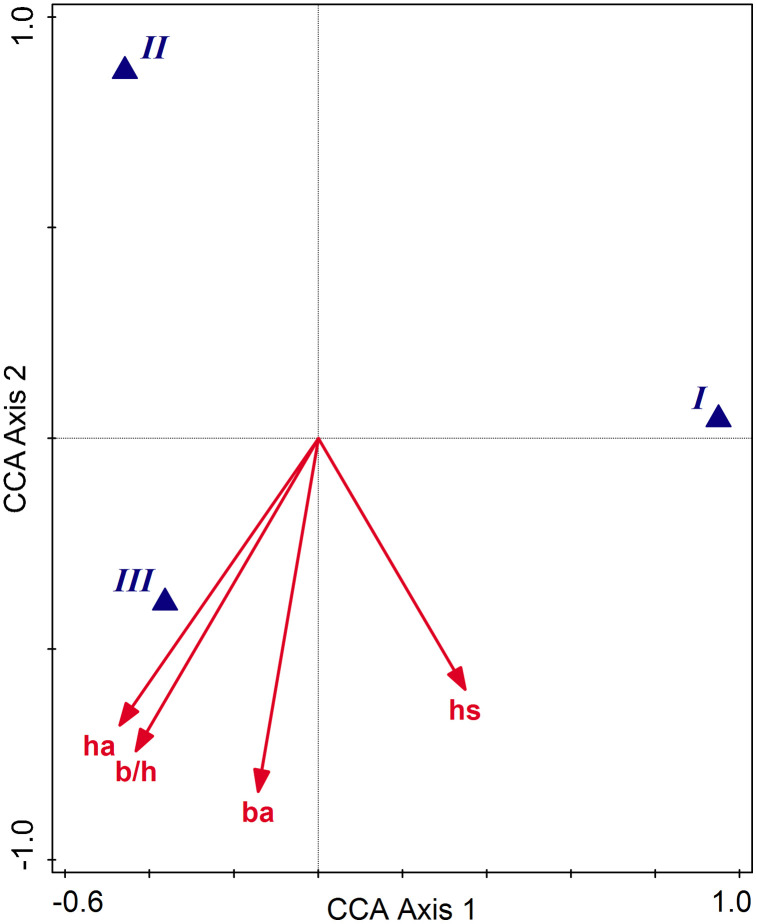
CCA ordination diagram with species predicted by selected biometric variables. The three species are distinctly placed in three quarters (subspaces) of the ordination space defined by the first two axes, most biometric variables increasing towards species **III.** Axis 1 clearly separates species III, while axis 2 discriminates species I and **II.**

**Fig 8:**
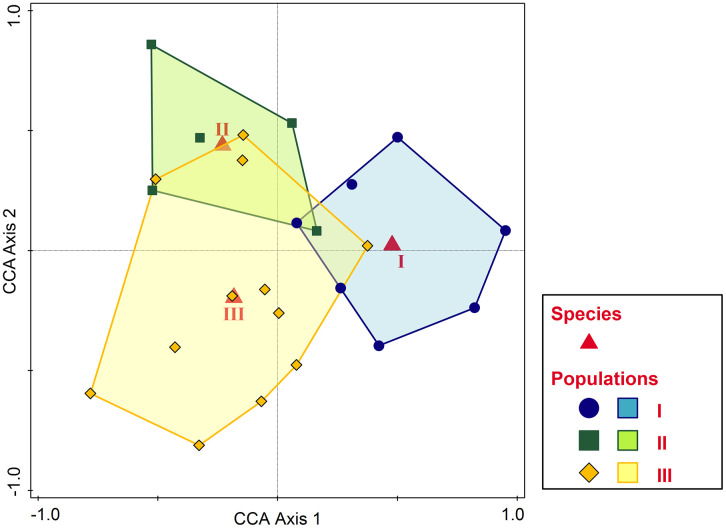
Classification diagram of populations into species in CCA ordination space constrained by selected biometric variables.

The CCA relationship between the species and the biometric variables was significant (test on all axes pseudo-F = 2.2, p = 0.029), the adjusted explained variation being 32.5%. The conditional test effects included four predictors, b/h, hs, ha, and ba, with marginally significant p-adj values. Except for hs, the biometric features are higher in species III, species I maximizes hs and displays average biometric values, while species II shows the lowest values for the morphological features (Fig 7). The classification diagram of populations into species and constrained by selected biometric predictors in CCA is given in Fig 8. Mostly distinct morphological populations represent species I and II in contrast to species III, which displays somehow intermediate characters. However, in all cases, the overlap is partial, and the unique (non-shared) areas prevail, pointing to a statistically significant morphological distinctness of the pseudocryptic species.

### 3.4. Classification into species related to p-distances

When species were considered as classes of objects and used in a db-RDA for predicting the variability in the p-distances, the adjusted explained variation was 87.4%, relations being highly significant (test on all axes, pseudo-F = 205, p = 0.001; Fig S3 in S1 Supporting Information). When species categories served as response variables and PCoA axes scores based on the p-distances were used as predictors, the CCA resulted in a highly significant relation (on all axes p = 0.001), the adjusted explained variation being 99.1%.

### 3.5. Classification into mOTU and species explained by climatic and spatial predictors

The 21 climatic predictors were responsible for 48% of the variation in classification into mOTU (pseudo-F = 3.6, p = 0.001 on all axes). Most climatic parameters had a significant simple term effect, the non-significant being some temperature values (Tavg, Toct, Tjul, and Tapr). Significant predictors in terms of conditional effects were Roct, Woct, RHum, Wjul, Tmax, Wapr, and Rjan, whereas Tno10 was marginally significant. The simple and conditional term effects are given in Table S3 in S1 Supporting Information.

**Fig 9:**
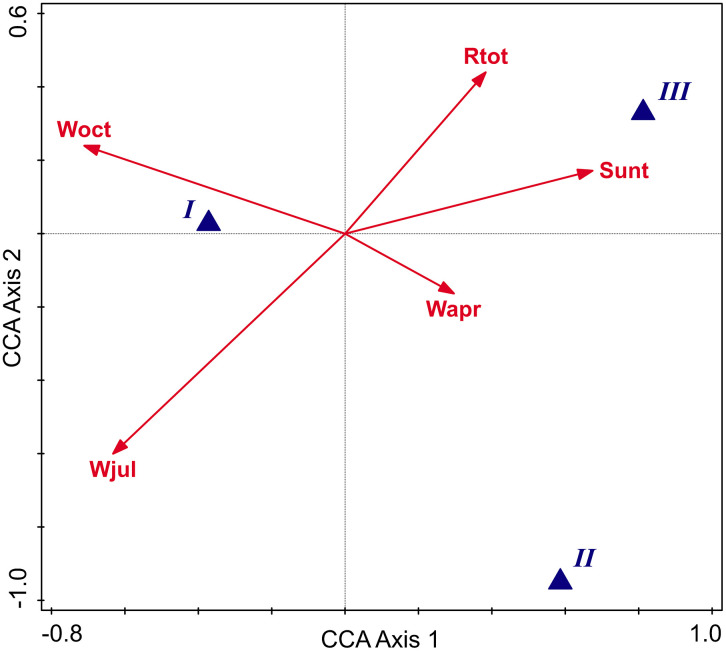
CCA diagram for species explained by selected climatic parameters (adjusted explained variation is 57.4%).

The adjusted explained variation in classification into species by climatic descriptors was 67.8% (on all axes pseudo-F = 6.9, p = 0.01). The significant conditional term effects (p < 0.05) were registered for four predictors related to humidity and precipitations and one linked to the duration of sunshine (Wjul, Woct, Wapr, Rtot, and Sunt). There are contrasting climatic preferences for the different species. Species II prefers areas with high values of wet days in spring; species I range is characterized by maximal values of wet days in summer and fall. In contrast, the species III range is characterized by maximal values of total amounts of annual precipitation and of average duration of annual sunshine in percent of possible (Fig 9).

We explored the relations between the classification of mOTU and spatial eigenfunctions, namely the PCoA axes scores from PCNM (or db-MEM), by CCA. The relation was marginally significant (test on all axes pseudo-F = 1.5, p = 0.052, R^2^-adj = 8.8%). The significant predictors (both in terms of simple and conditional effect) were the axes scores of PCoA2 and PCoA3, meaning functions related to larger-scale processes. By the same method, we also tested the ability of the spatial eigenvectors to discriminate between the three pseudocryptic species. The results show a highly significant relation (pseudo-F for all axes 2.5, p = 0.004, R^2^-adj = 21.9%). If only the adjusted p-value is considered then the single significant predictor of the species is PCoA3 (p-adj = 0.011), but according to the unadjusted values of conditional terms effects, next to the former axis, also the axes scores of PCoA2 (p = 0.023) and, marginally significant, of the PCoA1 (p = 0.052) are added. Larger scale events discriminate between these pseudocryptic species.

### 3.6. Other relations between the matrices

In an RDA of climatic response variables predicted by spatial polynomials, the adjusted explained variation was 84.7% (p = 0.001). All simple effects were significant, while the conditional effects were significant for Alt (r^2 ^= 40.8%), followed by X^3^ (r^2 ^= 36.3%), Y (r^2 ^= 5.7%), and XY (r^2 ^= 5.3%).

Testing the relation between the morphological data table and the PCoA axes scores of genetic distances resulted in a marginally significant (p = 0.089 for the first axis) model with R^2^ = 33%. Selecting only the predictors with conditional significant effects (PCoA axis 1, 2, 4, and 13), the model was significant (p = 0.004 for both the first and all axes), and the adjusted explained variation was 34.1%.

### 3.7. Variation partitioning between predictors of species discrimination

**Fig 10:**
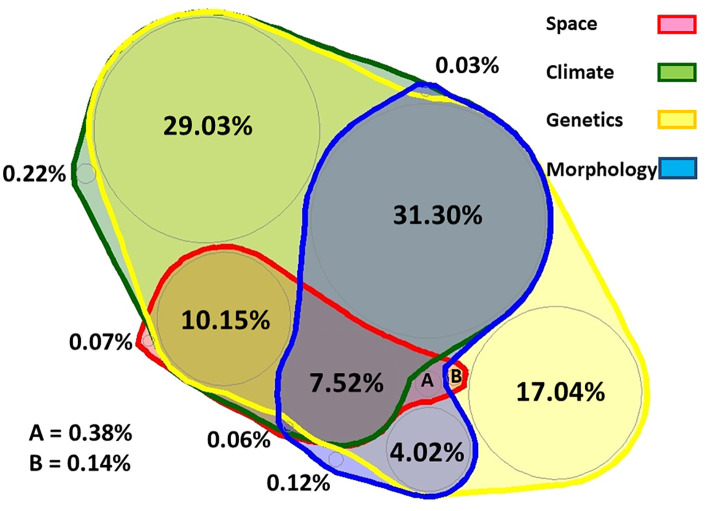
Variation partitioning diagram of the selected predictors from the categories describing the genetics, morphology, climate, and space in relation to species. The response variables were the three species, considered as binary dummy variables, and the method used was CCA. The values are percentages of un-adjusted variation explained by the selected predictors’ categories.

We tested, selected, and partitioned, by means of CCA, the capacity of descriptors belonging to all classes to predict the classification of populations to the three species. The selected predictors were the variables that had a significant explanatory power in the CCA relating the mOTU classification separately to each class of variables, namely b/h, hs, ha, and ba for morphology (0.05 < p < 0.1), Wjul, Woct, Wapr, Rjul, and Tmin for climate (p < 0.05 except for Tmin, which was marginally significant), PCoA3 axis scores for space (p = 0.007) and the first six PCoA axes scores for genetic p-distances (p < 0.05 except for PCoA4, which was marginally significant). Only the selected genetic predictors showed a larger unique effect (about 17%), the rest having unshared explained variation of less than 1%. As expected, because the three pseudocryptic species have been described by biochemical molecular characters, the genetic predictors explained about 99% of variation in species categories. However, most part is shared with other classes of predictors. Following the species discrimination by genetic features, the descending order of variation explained by the predictor classes is climate (78.2%, adjusted explained variation 72.1%), morphology (43.3%, adjusted value 31.4%), and spatial eigenfunctions (adjusted explained variation 14.5%). The main shared effects are between genetics and climate (29%), and among these and morphology (31.3%). All four classes of predictors have a shared effect of about 8% (Fig 10 and an alternative representation Fig S4 in S1 Supporting Information).

**Fig 11:**
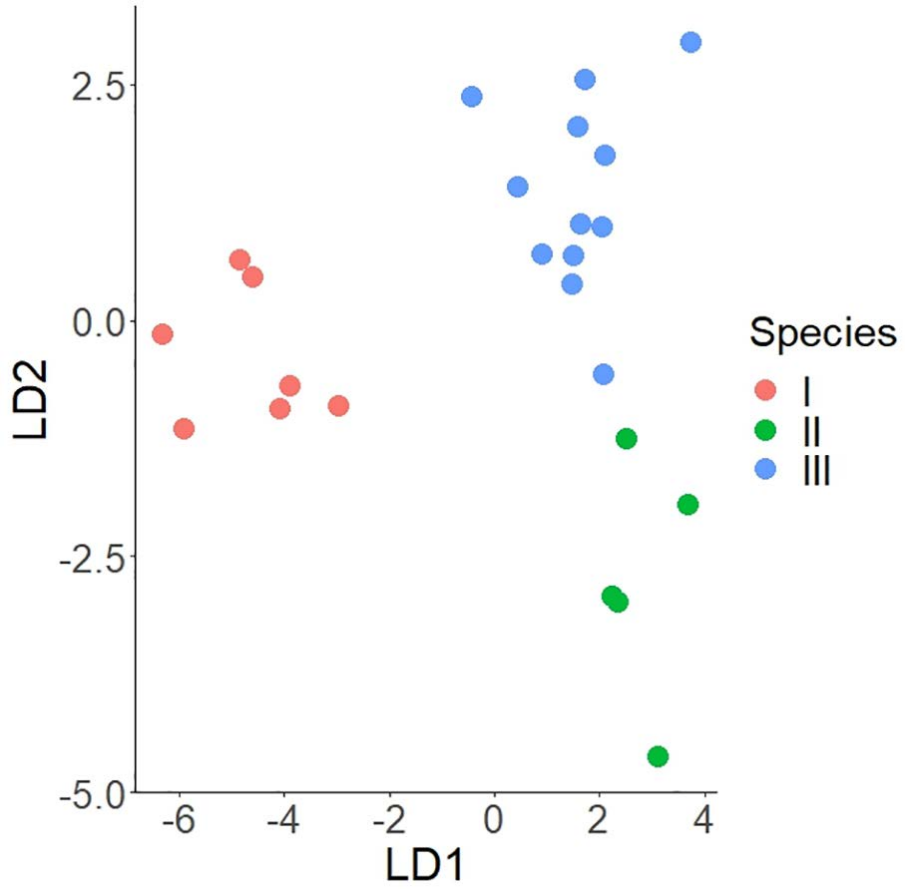
Linear Discriminant Analysis (LDA) plot, illustrating the segregation of the three species along the two discriminant axes.

We have applied an LDA for the selected predictors without the p-distances to test their capacity to discriminate between species. The deliberate discarding of genetic features (the PCoA axes scores resulted from the p-distances matrix) helped us understand the use of other features in recognizing and determining the cryptic species. The LDA based on ten selected predictors (four morphological, five climatic, and one spatial) proved to be highly significant (Wilks’ lambda = 0.022, F = 6.84, p < 0.0001), the classification matrix of species consisting of observed classification (on rows) by predicted classification (on columns) showed a 100% correct percent, namely a perfect classification of each species based on the samples in the model (Fig 11).

The most important predictors of species classification were related to climate, especially the number of wet days (Wjul, Woct). Considered as the single predictor, space (PCoA3S) also had a significant effect on the discrimination among species. However, because of the spatial patterns of climate, their effects overlap, and when climate is considered, the spatial partial effect is not significant (Table 2). Among the morphological variables, b/h and ha had a simple significant effect, but when climate was considered, only b/h had a marginally significant partial effect (Table 2). Delineation among the three species was significant along both discriminant axes, as shown by the one-way ANOVA results (F_2,21_ = 110.1, p < 0.001 for LDA1 and F_2,21_ = 30.5, p < 0.001 for LDA2)

**Table 2:** Overall and partial effects of predictors in the Linear Discriminant Analysis (LDA) of species classification based on climate, morphology, and space. Wilks’ lambda and the associated F and p values were calculated separately for each predictor (simple), for the models including the variables selected so far (overall), and for comparing the model including the new variable with the model not including it (partial).

Variable	simple			overall			partial	
	Wilks’ lambda	F	p	Wilks’ lambda	F	p	F	p
Wjul	0.606	6.81	0.005	0.606	6.81	0.005	6.81	0.005
Rjul	0.942	0.65	0.534	0.322	7.63	< 0.001	8.84	0.002
Tmin	0.655	5.53	0.012	0.185	8.40	< 0.001	7.05	0.005
b/h	0.716	4.16	0.030	0.137	7.64	< 0.001	3.11	0.068
Woct	0.612	6.66	0.006	0.104	7.14	< 0.001	2.71	0.094
Wapr	0.971	0.31	0.736	0.032	12.29	< 0.001	18.21	0.000
ha	0.727	3.94	0.035	0.029	10.51	< 0.001	0.81	0.464
ba	0.754	3.42	0.052	0.024	9.54	< 0.001	1.36	0.286
hs	0.817	2.36	0.119	0.023	8.15	< 0.001	0.39	0.687
PCoA3S	0.636	6.02	0.009	0.022	6.84	< 0.001	0.11	0.898

In the jackknife resampling cross-validation, the percentage of samples not seen by the model that were correctly classified was 79.2%. The most parsimonious model including Wjul, Rjul, Tmin, and b/h, classified correctly 87.5% of the samples in the model and 79.2% of the samples in the jackknife resampling cross-validation.

The LDA results prove that even when not described by genetic features, cryptic species might also be discriminated by a combination of other traits or taxonomic characters, like the selected predictors of climate, space, and morphology.

## 4. Discussion

In the present article, we examined the interplay between genetics, morphology, climate, and spatial descriptors and their effect on populations variability, mOTU, and cryptic species discrimination. We have shown that cryptic species may be statistically recognized and identified by a wide range of characters and features related not only to genetics but also morphology, climate, and space, presenting an example involving three pseudocryptic species of the *Fruticicola* genus.

*Fruticicola fruticum* was considered to have a wide geographic range in Europe, reaching from the Urals and the Caucasus to the Balkans and from the southern part of Scandinavia, through Central Europe to eastern and central France and northern Italy. However, it was revealed to be a complex of three pseudocryptic species [20]. Based on genetic evidence, two new species have been described, namely *F. similis* Proćków et Sîrbu, 2022, which currently is known only from Transylvania (central Romania), and *F. gemina* Proćków, 2022, ranging from central to south-western Romania, Bulgaria (Sarnena Gora and western Rhodopes Mts), Croatia (western Slavonia), Montenegro (Tara valley), west Serbia, to central Bosnia and Herzegovina [20]. Soon after the publication, the status of the newly described species *F. similis* was questioned (Ruud Bank pers. comm.) since earlier, a “subspecies” was described from a nearby area. Based on samples from an alder vegetation habitat in the Cumpătu Nature Reserve near Sinaia (Romania), Al. V. Grossu described on the basis of some morphological characters the subspecies *Bradybaena fruticum popovici-baznosanui* Grossu, 1980 [70]. Molecular analysis of individuals sampled by I. Sîrbu and A.M. Benedek from the *locus typicus* in July 2022 suggested that the two taxa may belong to the same species. Following the priority rule, the species *F. similis* Proćków et Sîrbu, 2022 should be considered a junior synonym of *Fruticicola popovicibaznosanui* (Grossu, 1980). The phylogeny appears to be more complex, with greater genetic variability within the species, and an extended analysis of the material and corresponding systematical revision will be published shortly.

In our study, all the biometric variables showed a significant decrease, either linear or quadratic, with latitude. This means that at higher latitudes, where mean temperatures are lower, the mean values of morphometrical variables are smaller. Thus, snails from southern populations tend to have larger and flatter shells. There are many observations on the proportions of the shells in the Helicoidea, describing proportionally higher spires at warmer localities; our data also suggest a correlation with humidity. Thus, species might be somehow discriminated by morphology, but they cannot be determined only by means of biometric characters. There is evidence that at least some biometric variables might discriminate the pseudocryptic species to a certain degree, or at least between some pairs. It is interesting that the biometric variables that discriminate between species are completely different from those that distinguish between the mOTUs. Species *F. fruticum* (I) and *F. popovicibaznosanui* (formerely described as *F. similis*, II) are clearly differentiated by biometric variables, while *F. gemina* (III) is partially and slightly overlapping with I and significantly overlapping with II. We conclude that biometric features discriminate better and more significantly between species than mOTUs, thus their ability to predict the taxa the snails belong to should not be underestimated.

Transitions of the shell shape, namely subglobose-globose-flat, occurred many times in the phylogeny of the Australian Camaenidae [71]. In two polygyrid land pulmonated snails, experiments examining the influence of various factors on shell form and growth rate [72], which determines the adult shell size, revealed that most variation (70%) in spire height is heritable and only 10% is induced by environment, humidity causing its increase. In contrast, the growth rate is 50% induced by the environment (temperature having twice the effect of humidity), and 30% heritable (within-cohort variation), whereas the genotype-environment interaction explained 10% in both traits. Gould [73] and Clarke *et al.* [36] also stressed the significant genetic component of shell variation. Another problem affecting the comparisons of the shell proportions between local populations is a result of the allometric growth of the gastropod shells [73]: higher (older) shells have relatively higher spires.

In our study, the geographic latitude gradient, causing the temperature decrease, was reflected by decreasing shell breadth, body whorl height and breadth, and proportion between the shell breadth and height; thus, the snails from southern populations had bigger and flatter shells. The geographic longitude was reflected by the higher spire and wider shell towards the West (with the increasing humidity). In general, the spatial predictors had less effect on the shell biometry than the climatic ones. However, contrary to the expectation based on the latitudinal and longitudinal trends in temperature and rainfall, their shared effect was comparatively small.

Among the climatic variables, we found the humidity (rainfall, wet days) to have the major effect on the shell size and morphology. The contrast between aridity and humidity appears to be a crucial factor controlling the shell biometry [40,74]. Cook and O’Donald [35] and Knights [38] found that in the European *Cepaea,* smaller snails survived better in unshaded or warmer conditions. *Helix pomatia* Linnaeus, 1758 and *H. aspersa* O. F. Müller, 1774 attain a slightly larger size when raised with chalk [42], whereas *F. fruticum* does not [75]. The variation in relative shell height within species correlates with environmental factors in several species [41]. The relative height increases with increasing rainfall in *Levantina spiriplana* (Olivier, 1801) in Israel [50] and *Cepaea nemoralis* (Linnaeus, 1758) in Belgium [37], and with increasing humidity of habitat in *Discus rotundatus* (O. F. Müller, 1774) in Germany [45], but in *Pleurodonte lucerna* (O. F. Müller, 1774) in Jamaica just opposite, with decreasing rainfall [49]. In miscellaneous helicid species in Europe the shell relative height increases with increasing elevation above the sea level [76]. Emberton [72] has reported that flatter shells “make better estivators (shelterers from drought)”. All examples of tropical snails and the European species *Discus rotundatus* show a negative correlation between relative height and rainfall, whereas the examples from Israel show the opposite pattern [41]. Drier conditions may select for more but narrower whorls, resulting in a smaller surface of the aperture [41].

Hayakaze and Chiba [77] studied the patterns of change in shell width over 35,000 years ago, based on fossil records of three Camaenidae species: *Euhadra pachya* (Pilsbry, 1902), *Phaeohelix phaeogramma* (Ancey, 1888), and *Coniglobus mercatorius daemonorus* (Pilsbry, 1902) on the island of Kikai in the Ryu-kyu Islands (south-western Japan). The shell width fluctuated through time synchronically in these species. The temporal variation in the fossil gastropods was larger than the geographical one among the extant populations. These temporal changes in the relative shell height were correlated with the patterns of climate change. In another species of the Camaenidae, *Mandarina polita* Chiba, 1989 from the Ogasawara Islands [47], shell morphology correlated with microhabitat—in wet and sheltered sites shells were high-spired, with a high aperture, while in dry and exposed sites they had low spire and broad mouth; the morphology was associated with the depth of the leaf litter.

Goodfriend [41], in his review of the shell form in land snails, reported that larger shells are often associated with increased humidity conditions. Similarly, Lazaridou-Dimitriadou *et al.* [78] in their study on the Greek *Helix aspersa*, reported that the aperture area and the largest shell diameter were both negatively correlated to the mean minimum annual monthly temperatures, suggesting that larger snails do not survive in warmer conditions, and the aperture—the main site of water loss [74]—relative area diminishes under drier conditions, to reduce water loss. Goodfriend [41] also reported that adults may be smaller in higher population densities.

In our study, the genetic distances were highly and significantly explained by the spatial gradients, the latitude being much more important (about 90% in relative terms) compared to longitude; the polynomial terms of coordinates explain about two-thirds of the maximum variation in p-distances between the populations. Compared to latitude, altitude played a minor role, but not an insignificant one if singularly considered, in explaining the genetic distances between the populations. Thus, when considered together with spatial coordinates, the unique effect of altitude is not important in explaining genetic p-distances between populations. The p-distances variability was explained by spatial (unique effect 4.7%), climatic (1.2%) and by both predictor groups (shared effect 77.4%), adding up to 83% adjusted explained variation.

Genetic similarity among distant populations may arise from various factors [79–81], but we observed [20] that the general pattern of interpopulation differentiation of *Fruticicola* approached neither isolation-by-distance nor even stepping-stone, but rather the infinite-island model [55,81]. However, we have found a linear association between genetic and geographic distances in the present study. The spatial isolation is the main factor shaping the genetic differences [36,53–55]. In general, genetic distances reflect the differences in the genotypes. Thus, the climatic factors, partly explaining the genetic distances, seem to reflect selection, acting differently in various localities. There are several publications demonstrating the selection acting on the shell quantitative characters in the land pulmonates [35,36,38,40,49].

Differences in the climatic preferences have been observed between the cryptic species (although they occurred in allopatry, thus climatic parameters *per se* might not be excluded). This may confirm the biological distinctness of these species, which have been distinguished only by molecular data so far. Minute morphological differences between cryptic and semicryptic species are reflected neither in minor differences in biology nor in the less distinctness of a species as such. Similar conclusions reported [12,13,15–19].

Comparing the capacity of predictors to ascribe populations to either mOTUs or species, the explained variation and implicitly the predictive abilities are higher in the latter. Biometric explanatory variables explain classification in mOTUs by R^2^ = 22.1%, while the corresponding value for species is 32.5%. Correspondingly, climatic predictors account for 48% of the classification of mOTUs and 67.8% of species, whereas spatial eigenfunctions explain 8.8% (marginally significant) of mOTU and 21.9% of species differentiation. Thus, using biometric, climate, and spatial eigenfunctions criteria leads to a better prognosis when mOTUs are grouped into specific taxa.

The final variation partitioning analysis (Fig 10, and its alternative Fig S4 in S1 Supporting Information) quantifies the relative contribution of genetic, morphologic, climatic, and spatial predictors in evaluating the extent to which each category explains variation among the three cryptic species, treated as binary response variables. The percentages of explained variation offer insights into the evolutionary and ecological processes that have shaped species differentiation. The relative explanatory contribution decreases from genetics to climate, morphology, and spatial predictors. Space is the least notable among the predictors, possibly due to dispersal mechanisms and barriers. The single larger value of the unique effect is registered for the genetic predictors, with all the other categories showing small singular effects (non-shared explained variation), indicating a strong genetic basis for their variation. The intersection (shared explained variation) of the four predictor groups is small (7.5%) meaning that although they interact, each explanatory category has a certain independent contribution. Morphological variability is linked mainly to genetics and (secondary) to climate and, to a lesser degree, to space. The relatively low interaction of climate and space suggests that dispersal limitations have a minor role in species delineation and distribution. Morphological variation expressed in species delimitation (and consequently divergence) is less linked only to genetic factors (about 4%), but more to the shared effect of genetics and climate (31%), indicating a mediated consequence of genetic differentiation rather than a direct response to environmental conditions.

In our study on the variability and discrimination of cryptic species by integrating systematics with morphology, genetics, climate, and spatial descriptors, we have shown that each of these predictor categories has its share of significance. However, the study has its limitations, mainly related to the small sample size (24 samples) for the biometric data, which can hardly describe the three species. It is likely that in the future, adding new morphological data from other populations may increase the quality of the models and their predictive power, bringing evidence to the problem of discriminating between the species by means of shell variability.

It might be considered that the procedure outlined in our work is complex and genetic-based alternatives, including barcoding, are preferable in identifying taxonomical units. Barcoding and any genetic method aimed at determining the taxon is like asking for somebody’s name. It is the answer to the question of ‘who’ or a very bare identity. When answering questions about someone’s profession, status, or functions, we are asking, ‘What and how is that entity doing, and how is it related to its environment?’. Identifying taxa through an interplay between genetics, climate, spatial descriptors, morphology, and possibly others enables the characterization of the taxon in terms of its adaptations, ecology, and more, thereby broadening the perspective. Its status is no longer just a bare identity but features of its ecological niche, how it relates to the environment, and the outcome of its evolution. In the end, our approach is not to replace but to methodologically broaden and complement modern systematics and its use.

In future investigations, other classes of explanatory variables (e.g., habitat types and their features such as vegetation, soil, and land use) should be included, and their effects must be assessed and tested, both as unique and shared, in addition to those already used in this article. By these, discrimination between cryptic species and the mechanisms of their evolution might be better understood and used in further studies. We conclude that further research should investigate the importance of additional ecological drivers, such as relations with other predictors not included in this study and microhabitat specialization, in shaping species delimitation and distributions. Additionally, incorporating whole-genome sequencing could provide deeper insights into adaptive genetic variation, its relationship to morphological traits, and their responses to environmental gradients.

## 5. Conclusions

The traditional cryptic species classification and further identification using genetic data can be expanded within a multidisciplinary framework that also uses morphology, climate, spatial descriptors, and possibly others. We show that although cryptic species are described based on genetic features, climate, morphology, and space predictors can clearly discriminate between these, in our case, the LDA proved a 100% correctness of the model. This provides significant support for field researchers when the genetic analysis is difficult or impossible to apply when sampling and preserving biological material is not wanted or is undesirable, in ecological studies (at both population and community levels), and in many other contexts. As such, our contribution has practical implications, highlighting the use of multivariate canonical analysis in identifying and distinguishing cryptic species, something particularly relevant for field researchers and ecological studies where genetic testing is impractical and might pose far-reaching effects for conservation and legislation. It also brings more insight into species variability analysis and mechanisms of speciation by linking phylogeny to genetic heterogeneity and distances and further to morphology, climate, and spatial patterns, linking mathematical models aided by IT to biology and ecology. To understand evolution and particularly speciation, new methods of multivariate and multi-matrix analysis and related illustrative techniques have to be developed and applied in future studies. It also hints towards the need for revised taxonomic approaches, including but not limited to established criteria, such as genetic and morphological methods, while integrating spatial, climatic, and other environmental descriptors, which might further lead to a more functional and integrative species delineation. As such, contributing to a more detailed understanding of species diversity and using a more integrative and holistic methodological framework of taxonomy will enhance systematics, evolutionism, and ecology in theory and their applications.

## Supporting information

S1 Supporting Information Supplementary figures and tables. (PDF)
