# Supplementary material for: Delineation in fuzzy boundaries: Canonical ordination analysis discriminates between cryptic species by linking taxonomy to genetics, morphology, climate, and space
Source: PLoS One. 2025 Oct 31;20(10):e0334617. doi: 10.1371/journal.pone.0334617 (PMC12578234; doi:10.1371/journal.pone.0334617)
Supplement: S1 Supporting Information — (PDF) [file pone.0334617.s001.pdf]

Supporting Information

Delineation in fuzzy boundaries: canonical ordination analysis discriminates between cryptic species by linking taxonomy to genetics, morphology, climate, and space

Ioan Sîrbu[1,2*], Ana Maria Benedek[1*], Sebastian Hofman[3], Aleksandra Jaszczyńska[4,5], Andrzej Falniowski[4]

[1] Biology and Ecology Research Center, Faculty of Sciences, Lucian Blaga University of Sibiu, Sibiu, Romania
[2] Doctoral School of Engineering Sciences and Mathematics, Lucian Blaga University of Sibiu, Sibiu, Romania
[3] Department of Comparative Anatomy, Institute of Zoology and Biomedical Research, Jagiellonian University, Kraków, Poland
[4] Department of Malacology, Institute of Zoology and Biomedical Research, Jagiellonian University, Kraków, Poland
[5] Department of Invertebrate Evolution, Institute of Zoology and Biomedical Research, Jagiellonian University, Kraków, Poland

* Corresponding authors
E-mail: ioan.n.sirbu@ulbsibiu.ro (IS), ana.benedek@ulbsibiu.ro (AMB)

Table S1. Statistics of the additive models (AM) relations between shell morphology (biometrical descriptors) and latitude. Codes of morphological variables are explained in the text.

AM fitted for nine response variables:				
Response	Type	R^2 (%)	F	p
b	quadratic	38.4	6.5	0.006
ba	quadratic	25.6	3.6	0.045
bw	linear	28.3	8.7	0.007
h	quadratic	26.7	3.8	0.038
hb	quadratic	36.8	6.1	0.008
ha	linear	29	9	0.007
hs	quadratic	20.8	2.8	0.086
pw	quadratic	25.9	3.7	0.043
b/h	linear	27.8	8.5	0.008

Table S2. Statistics of the additive models (AM) relations between shell morphology (biometrical descriptors) and longitude. Codes of morphological variables are explained in the text.

AM fitted for two response variables:				
Response	Type	R^2 (%)	F	p
hs	linear	15.8	4.1	0.05406
b/h	linear	12.1	3	0.09527

Table S3. Post-hoc simple and conditional term effects of classification into mOTU explained by climate predictors. The codes are described in text.

Simple Term Effects:					Conditional Term Effects:				
Name	Explains %	pseudo-F	P	P(adj)	Name	Explains %	pseudo-F	P	P(adj)
Roct	12	7.9	0.001	0.00175	Roct	12	7.9	0.001	0.007
Woct	11.1	7.3	0.001	0.00175	Woct	11.3	8.4	0.001	0.007
Rapr	11.1	7.2	0.001	0.00175	RHum	8.9	7.3	0.001	0.007
RHum	10.4	6.7	0.001	0.00175	Tno10	3.5	3	0.03	0.07875
Rjan	10.2	6.6	0.001	0.00175	Wjul	4.1	3.7	0.002	0.0105
Wjul	10	6.4	0.001	0.00175	Tmax	4.1	3.8	0.004	0.0168
Wet	9.5	6.1	0.001	0.00175	Toct	3.4	3.4	0.052	0.12133
Wjan	9.1	5.8	0.001	0.00175	Wapr	3.2	3.3	0.016	0.048
Rtot	8.9	5.7	0.001	0.00175	Rjan	3.2	3.5	0.008	0.028
Rcv	8.3	5.3	0.001	0.00175	Wjan	1.5	1.7	0.127	0.24245
Sunt	8.3	5.3	0.001	0.00175	Rtot	1.3	1.4	0.223	0.3345
Wapr	7.5	4.7	0.001	0.00175	Rapr	1.5	1.7	0.153	0.26775
Tmax	4.4	2.7	0.019	0.0285	Tjul	1.3	1.5	0.205	0.33115
Tno10	4.3	2.6	0.021	0.0294	Sunt	1.1	1.2	0.305	0.37676
Rjul	4.1	2.5	0.019	0.0285	Wet	1.8	2.1	0.086	0.1806
Tmin	3.8	2.3	0.03	0.03938	Rcv	1.1	1.3	0.252	0.3465
Tjan	3.6	2.1	0.062	0.07659	Tapr	1.1	1.3	0.264	0.3465
Tavg	3.2	1.9	0.091	0.10617	Rjul	0.6	0.7	0.615	0.64575
Toct	2.6	1.5	0.16	0.17684	Tavg	0.5	0.6	0.535	0.59132
Tjul	2.1	1.2	0.268	0.2814	Tmin	0.3	0.3	0.846	0.846
Tapr	1.7	1	0.39	0.39	Tjan	0.8	0.9	0.49	0.57167

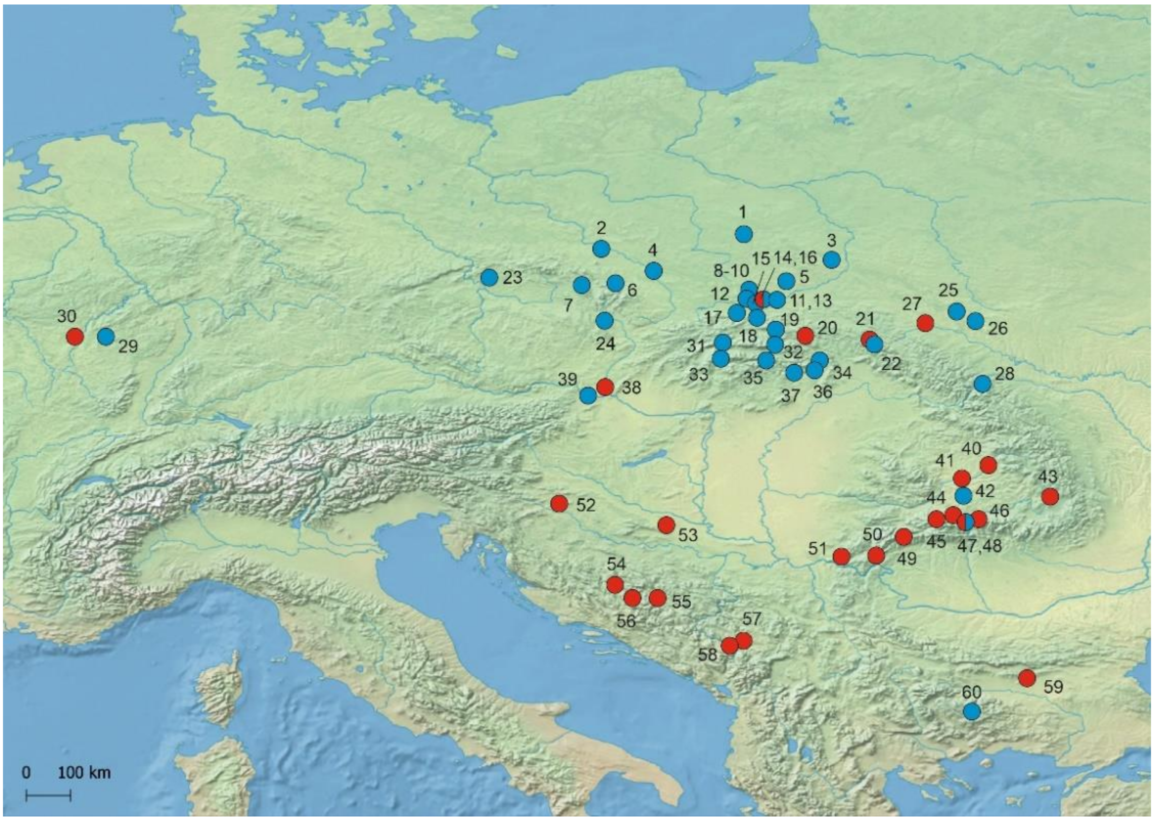

Figure S1. Map of *Fruticicola spp.* sampling sites with numeric codes corresponding to the database uploaded on the Figshare repository [1,2]. Red dots correspond to samples for which both genetic and morphological analyses were performed. The base map is from the Natural Earth public domain map dataset [3].

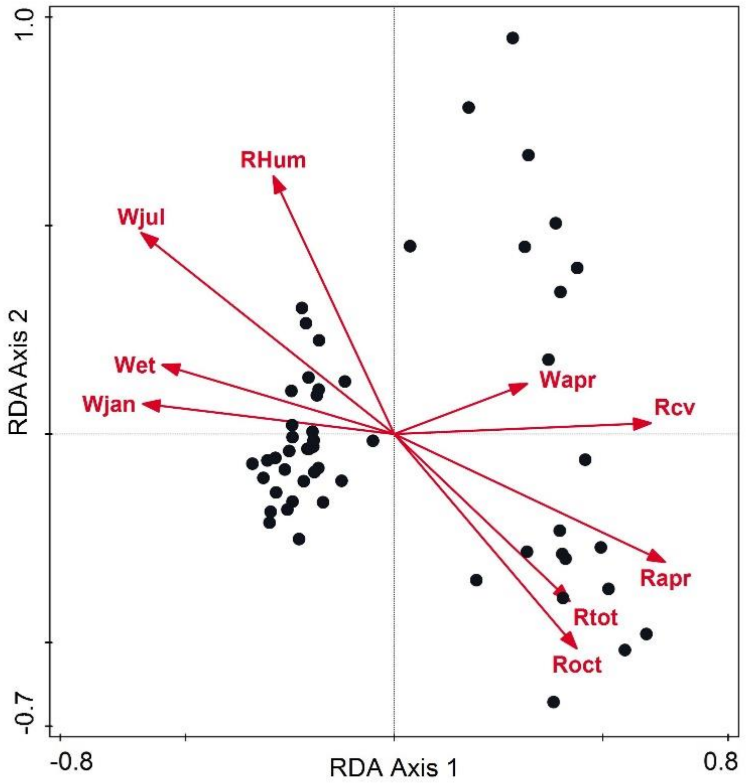

Figure S2. Distance-based redundancy analysis (db-RDA) ordination diagram between the p-distances of populations (projected in the ordination space as axes scores from a principal coordinates analysis—PCoA) and selected climatic parameters, by their conditional term effects. Codes of climatic variables (represented by arrows) are explained in the text.

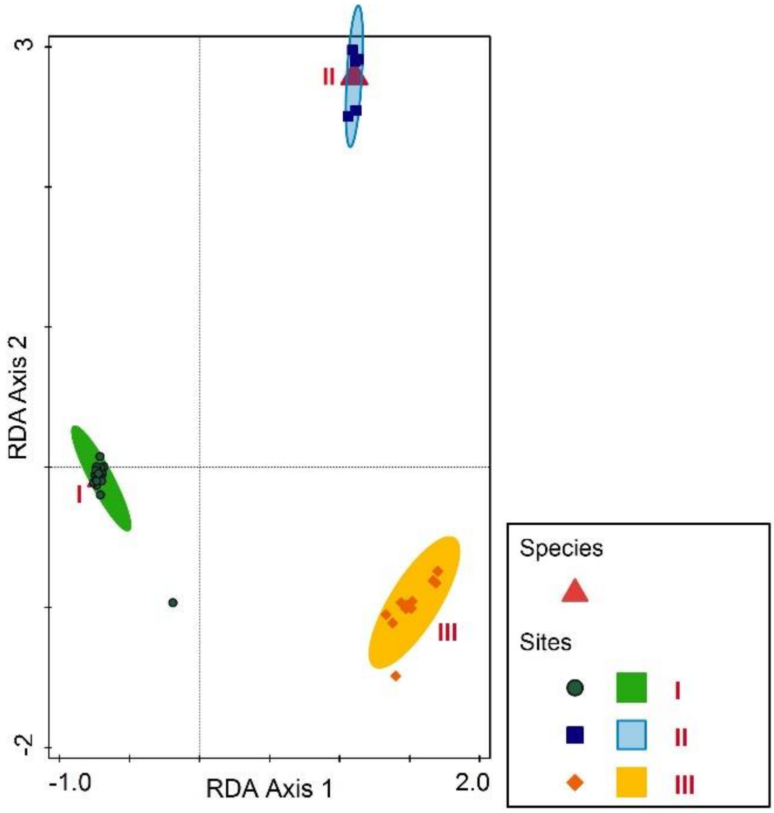

Figure S3. Distance-based redundancy analysis (db-RDA) ordination diagrams of p-distances explained by species.

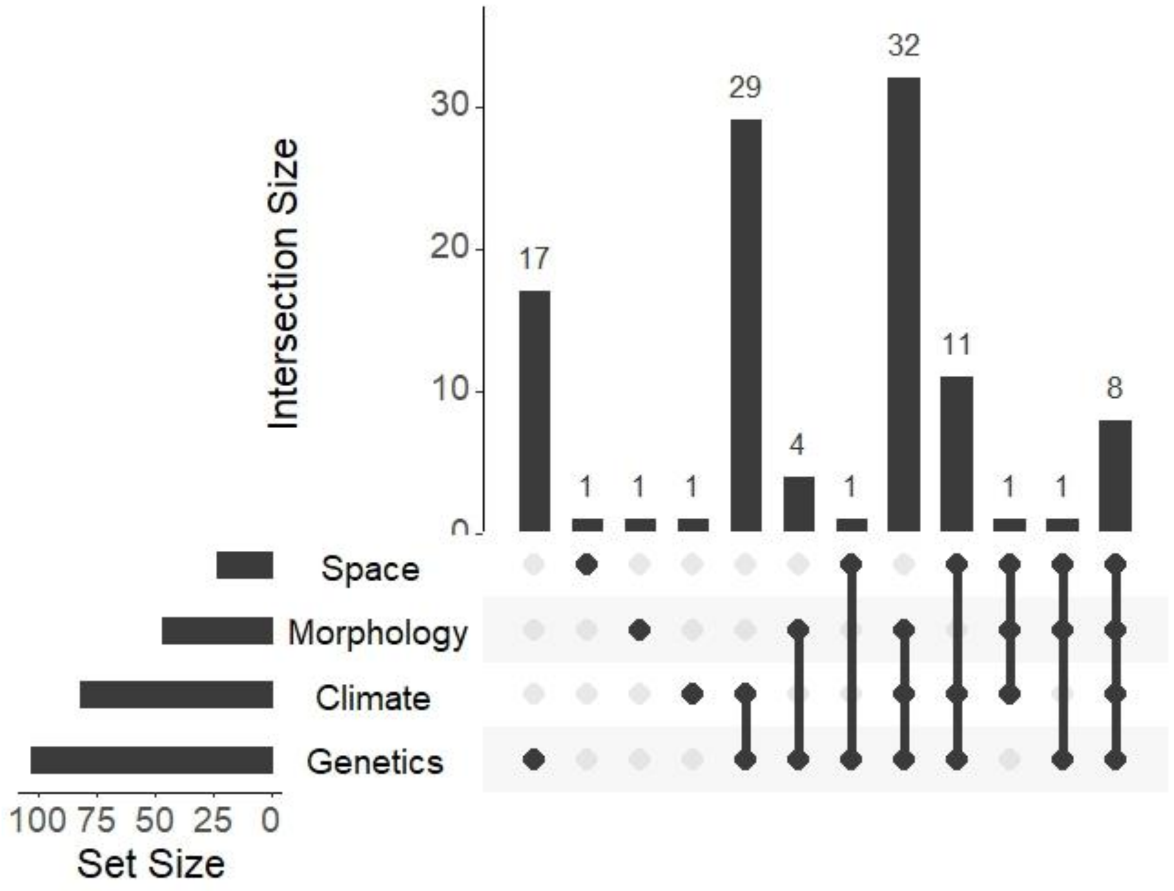

Figure S4. UpSet plot illustrating the results of the variation partitioning of the selected predictors from the categories describing the genetics, morphology, climate, and space in relation to species. Horizontal bars represent the total variation explained by the category of predictors, and the vertical bars represent the overlap between the categories indicated by the points. The values represent percentages of unadjusted variation explained by the selected predictors' categories and are rounded up to the closest integer.

References

1. Hofman S, Proćków M, Osikowski A, Jaszczyńska A, Sokół M, Sîrbu I, Benedek AM, Falniowski A. Data on distribution and variability of three pseudocryptic species: *Fruticicola fruticum* (O.F. Müller, 1774), *F. similis* Proćków et Sîrbu, 2022, and *F. gemina* Proćków, 2022 in Europe. 2025. https://doi.org/10.6084/m9.figshare.29828633"

2. Hofman S, Cameron RAD, Proćków M, Sîrbu I, Osikowski A, Jaszczyńska A, et al. Two new pseudocryptic species in the medium-sized common European land snails, *Fruticicola* Held, 1838; as a result of phylogeographic analysis of *Fruticicola fruticum* (O. F. Müller, 1774) (Gastropoda: Helicoidea: Camaenidae). Mol Phylogenet Evol. 2022; 168, article 107402: 1–18.

3. Natural Earth. Free vector and raster map data. https://www.naturalearthdata. com. 2025. Accessed 19 April 2025.
